# A Decision Support Framework for Periprosthetic Joint Infection Treatment: A Cost-Effectiveness Analysis Using Two Modeling Approaches †

**DOI:** 10.3390/jpm12081216

**Published:** 2022-07-26

**Authors:** Vasiliy N. Leonenko, Yulia E. Kaliberda, Yulia V. Muravyova, Vasiliy A. Artyukh

**Affiliations:** 1 Faculty of Digital Transformation, ITMO University, Kronverksky Pr. 49A, 197101 St. Petersburg, Russia; julia.kaliberda@gmail.com; 2 Russian Scientific Research Institute of Traumatology and Orthopedics Named after R.R. Vreden, Akademika Baykova St., 8, 195427 St. Petersburg, Russia; julia-muraveva@yandex.ru (Y.V.M.); artyukhva@mail.ru (V.A.A.)

**Keywords:** Markov model, periprosthetic joint infection, revision arthroplasty, total hip replacement, decision trees

## Abstract

Today, periprosthetic joint infection (PJI) is one of the leading indications for revision surgery and the most ominous complication in artificial joint patients. The current state of the art for treating PJI requires the development of methods for planning the costs at different scales to facilitate the selection of the best treatment methods. In this paper, we perform a cost-effectiveness assessment for strategies related to the treatment of PJI using a composite decision support modeling framework. Within the framework, two models are implemented: a detailed discrete-event probabilistic model based on the decision tree approach and a dynamic Markov model with generalized states. The application of the framework is demonstrated on the dataset which was provided by the Russian Scientific Research Institute of Traumatology and Orthopedics named after R.R. Vreden. The analyzed dataset contains 600 patient records divided into two groups (retrospective group, based on old records, and prospective group, based on real-time follow-up). The cost-effectiveness of treatment methods was compared based on associated costs and QALY units gained, with the mentioned two indicators calculated using two models independently from each other. As a result, two comparative rankings of cost-effectiveness of PJI treatment methods were presented based on the model output.

## 1. Introduction

### 1.1. Periprosthetic Joint Infection

Due to the advancement of public health and medicine, we see a stable increase in life span throughout the world. As a consequence, the diseases of the elderly are becoming more widespread and require more attention. Particularly, joint arthroplasty, also known as joint replacement, aimed at restoring the function of joints, is increasingly performed. The replacement of the affected joints with artificial ones creates a need for revision arthroplasty. Today, periprosthetic joint infection (PJI) is one of the leading indications for revision surgery and the most ominous complication in artificial joint patients [1,2]. Periprosthetic infection is associated with high morbidity, because the implant, as a foreign body, increases the pathogenicity of bacteria and the presence of a biofilm makes the diagnosis and treatment problematic [3]. As a result, complex strategies are required to treat PJI, including multiple surgical revisions and long-term antimicrobial treatment. The widespread use of antibiotics, the enhanced technical equipment of operating rooms and the development of surgical techniques made it possible to reduce the number of infectious complications from 9% in the early years of arthroplasty to 1.25% in recent decades [4]. Nevertheless, the number of cases of PJI is expected to increase in the near future and will double by 2030 [5]. It is also worth mentioning that the cost of PJI treatment is several times higher than of the primary endoprosthesis and places a significant financial burden both on the family budget of patients and on the state health care system. In 2005, the cost of revision hip arthroplasty related to PJI in the United States was 2.8 times higher than the revision arthroplasty related to aseptic instability and 4.8 times more expensive than primary total hip replacement [6]. In addition, the researchers found an increase in the cost of revision arthroplasty for infectious complications between 1997 and 2006.

During a large period of time, there was no firm, generally accepted understanding of PJI in the orthopedic community and supervising healthcare structures. Therefore, it was not possible to interpret data on the number of disease cases, the variants of disease course, the outcomes, and the economic effect of treatment strategies. The situation changed in 2011, when the Musculoskeletal Infection Society (MSIS) PJI Diagnostic Standards were first proposed [7]. Based on the analysis of the dynamics of changes in the modern theory of PJI, Haddad F. et al. believe that research in the next decade will be critical to further develop the understanding of PJI and to establish the most cost-effective treatment methods [4].

### 1.2. PJI Treatment Methods

Currently, a number of researchers consider the two-stage revision total joint replacement method as the most effective from the infection eradication point of view [3,8]. The leading indications for a two-stage revision total joint replacement (re-TJR) are cases of PJI, where the pathogen was not identified before surgery, the microflora is highly resistant, and there are bone or soft tissue defects [9,10]. Among the unsolved problems of two-stage re-TJR are: high costs, long period of disability, necessity of prolonged antibiotic therapy, blood loss, dislocation of spacers, and high mortality among patients between the stages [11].

An alternative method of treating PJI is the one-stage re-TJR. The long-standing discussion about the role and effectiveness of one-stage re-TJR in the treatment of chronic PJI is now almost overcome. The end of the discussion was initiated by the publication Zahar A. et al. in 2019, in which the authors reported long-term (10-year) results of a one-stage revision total hip arthroplasty (re-THR) [12]. They found that 94% of patients did not have a recurrence of PJI, 75% of patients did not require re-THR, and good and excellent functional results were found in 57% of cases. In studies with patients carefully selected before surgery, the success rate of one-stage re-THR was even higher, up to 100% [13].

Another possible way to improve the results of treatment with PJI is to shorten the time interval between the re-TJR stages. According to the results of the discussion in the International Consensus Meeting in 2018, it was found that there were currently no data indicating the optimal time interval between the stages of re-EP. Nowadays, the waiting period between operations can range from a week to several years. Most surgeons consider the period after wound healing, the end of antibiotic therapy, and the appearance of data on the positive dynamics of serological markers the optimal time for reimplantation [14]. However, the recommended time interval between re-EP stages is still unclear.

Last but not least, up to this date the experience related to re-TJR with partial preservation of stable endoprosthesis structures is still accumulating. Despite the fact that nowadays there are no generally accepted recommendations, in some cases, partial re-TJR can be the best alternative as it allows to avoid significant bone defects, reduce the degree of surgical aggression and problems with the choice of components of the endoprosthesis in the future [15]. Researchers also emphasize the importance of careful patient selection for partial re-TJR [16,17].

### 1.3. Cost-Effectiveness Analysis

It is known that the decision of surgeons to choose the method of treatment for individual patients with PJI is increasingly dependent on economic considerations, which is constraining and often not optimal [18]. Moreover, the cost of re-THR, missing generally accepted standards, differs significantly in national health systems. In 2012–2013, e.g., a two-stage re-THR for each case of PJI cost USD 90–100,000 in the USA [19], USD 57,000 (GDP 44,000) in the UK [5], and USD 53,000 (AUD 70,000 ) in Australia [20]. The current state of the art treating PJI requires systematization of costs and the development of methods for planning the costs at different scales—for separate individuals, on the level of healthcare units, and on the city level.

A state-of-the-art approach to medical practice requires justification of efficacy and safety of treatment methods. The methods of treating PJI should be selected based on evidence-based medicine, the means of which allow comparison, generalization, and wide practical application of the data obtained [21]. Thus, the need for a clinical and economic analysis of PJI treatment from the standpoint of evidence-based medicine seems to be very relevant, especially due to the lack of corresponding quality studies [22,23].

In addition to the problem of finding the best PJI treatment method in general, relying on both direct (a chance of successful PJI elimination) and indirect treatment outcomes (such as resulting increase in quality of life of the patients who underwent the treatment), there is an arising challenge of finding an optimal treatment strategy in advance for the particular patient based on his individual characteristics [24]. This challenge became an actual part of the personalized medicine concept and requires research based on a multidisciplinary approach, relying on statistical analysis, mathematical modeling, and machine learning [25].

### 1.4. Problem Statement

In this paper, we present a framework which compares the cost-effectiveness of PJI treatment methods. The patient dataset used in the study was provided by Russian Scientific Research Institute of Traumatology and Orthopedics named after R.R. Vreden, it contains records of the patients with PJI after total hip arthroplasty (THA). The methods of the cost-effectiveness analysis of treatment strategies are discussed and the method comparison is performed based on associated costs and QALY units gained. The ultimate objective of that direction of research consists in developing a computational tool to predict the consequences of a fixed treatment strategy for a given patient. Such a tool, when applied by healthcare professionals, will help enhancing the quality of PJI treatment both in terms of cost-effectiveness and the quality of life of individuals undergoing treatment.

## 2. Related Works

One study with objectives similar to ours has been published in [26]. The overall goal of the research was to estimate the effect of PJI treatment using contemporary treatment methods. The model of a hypothetical patient of working age with a PJI after a re-THR was built as a Markov state-transition model with the help of TreeAge Pro 2009 software. The algorithm accounts for a fixed percentage of patients undergoing one of three initial treatments: irrigation and debridement, single-stage exchange, or two-stage exchange based on available studies of current practices. The outcomes of these treatments were defined as septic/aseptic failures or successes. In addition, age-specific yearly mortality rates were used to predict transitions between the states. Patients who received successful treatment according to the model entered a state of being healthy which they might leave with a fixed annual rate related to repeated septic failure. Patients with failed treatment due to sepsis undergo a second procedure (two-stage exchange). The output of the model contains incidence and cost estimates associated with common medical complications. Each health state is assigned a cost for a fixed period of time (1 year). Transition probabilities determine the likelihood that a patient will either transition to the next health state or remain in the current one. For cost estimation by different rates of THA reinfection, the authors conducted a one-way sensitivity analysis (during the first year of treatment and beyond the first year). The main outcome of the research was to demonstrate that accounting for indirect costs and failures of treatment options dramatically increases the estimated overall treatment costs of PJI after total hip replacement. Thus, it seems to be much higher than it was thought in similar previous studies.

An example of a specific economic analysis based on data from one particular setting was presented in [27], where researchers considered the problem of PJI in low-middle-income countries. The aim of the study was to evaluate the incidence and economic burden of PJIs in a university hospital in Turkey. The costs in the model were set according to the information from hospital’s accounting system and included, among others, the cost of antibiotics, laboratory, radiology, prosthesis, operation, and total bed stay.

The research connected with PJI after Total Elbow Arthroplasty (TEA) was observed in [28]. The study aimed at investigating the risk factors which are associated with periprosthetic elbow infection, the incidence of infection after TEA and the acuteness of these infections. The importance of the study lies in the fact that compared to the knowledge regarding hip, knee, and shoulder PJI, research on prosthetic elbow infections is very limited and as a rule relies on data drawn from small case series. Authors used frequency tables to calculate the incidence of PJI among patients undergoing TEA. Frequency tables were also used to describe the details of patient presentation and management during admission for PJI. A logistic regression was used for each variable to determine the significance of each demographic variable as an independent predictor of PJI following TEA. As a result of the study, additional prognostic data were presented which, as the authors stated, could be used for patient selection and risk profile analysis.

A comprehensive review of the publications about PJI treatment cost-effectiveness analysis was performed in [22]. The aim of the researchers was to report the less costly and more effective procedures. The study highlighted existing problems in finding the best treatment method due to ambiguity of assessment techniques and data uncertainty.

The closest to the presented work is the article [29], where the investigators compare the effectiveness of 1-stage and 2-stage strategies for Total Knee Revision. Decision trees were built and subsequent Monte-Carlo simulations were used to calculate QALYs and costs. Sensitivity analysis was also performed to measure the influence of particular parameters on the outcome. Compared to this study, our research has an advantage of using unique patient data, while in the paper [29] all the parameters are taken from open sources.

## 3. Methods

### 3.1. Data

The analyzed dataset contains the records of patients who were subjected to revision total hip replacement (re-THR) in the period of 2000–2020. The patient records were collected in two different ways.

The first part of the disease histories was taken from the archives. The corresponding patient group is named ‘the retrospective group’. Initially the group contained 603 patients with PJI. The collection of information was started by personal examination and questioning of patients in the polyclinic of the Vreden’s Russian Scientific Research Institute of Traumatology and Orthopedics (25 (4.1%) patients). Patients who did not have the opportunity to come for examination were interviewed by phone (356 (59.03%) cases). In a number of observations, information about the condition of patients was obtained as a result of correspondence by mail (53 (8.8%) observations). In 169 (28.02%) cases, it was not possible to establish the results of PJI treatment and they were excluded from the study. The final list of patients in the retrospective part contained 434 patients with chronic PJI.

The prospective group of records included 166 patients with chronic PJI who were treated in the Department of Purulent Surgery of Vreden’s Russian Scientific Research Institute of Traumatology and Orthopedics in the period from 2016 to 2020. The record list contains those patients who were not subjected to exclusion from the study. Among the criteria of the latter were the following:

- Systemic inflammatory response syndrome, sepsis;
- Infectious inflammation of soft tissues of an unlimited form (phlegmon) or extensive purulent streaks to the neurovascular bundles;
- A soft tissue defect that does not allow the wound to be sutured;
- Implant-associated osteomyelitis IV (diffuse) anatomical type, patient’s physiological class C (Cierny-Mader classification);
- Recurrent course of PJI, when the number of reEP with implantation of an antibacterial spacer was equal of more than 3;
- Defects of the acetabulum not less than 3B and of the femur not less than 4 according to Paprosky classification, which were identified before surgery or formed as a result of surgical treatment.

All the patients were being observed for the possible PJI relapse till the end of 2020. In the retrospective group, the following treatment methods were applied: resection arthroplasty (RA), revision operation with the preservation of endoprosthesis (re-THR-PE) and two-stage revision total hip replacement with the two consecutive interventions separated by more than 2 months.

In the prospective group, new treatment methods were presented, namely, one-stage re-THR and partial re-THR (both 1-stage and 2-stage). The patients in the prospective group who underwent two-stage re-THR were divided into two subgroups based on their waiting time: 2–3 weeks and 6–8 weeks correspondingly.

The quantities of groups of patients of a certain age and gender in the records database are presented in Table 1. The description of treatment methods regarded in this study is presented in Table 2. As it can be seen the group ‘1-stage retro’ has extremely small sample size. Due to that reason it was excluded from this study.

Each patient record in the dataset contains their ID, birthdate, dates of registered health issues (manifestation dates), operation dates, types, and costs, the resulting state of the patient measured during his/her last attendance to healthcare services (PJI relapse or no PJI), and death date, if the patient died.

In case of the optimal treatment outcome, the resulting number of operations performed on each patient is defined solely by the PJI treatment method (for instance, two-stage re-THR assumes two interventions, with the installation of antibiotic-impregnated cement spacer and its subsequent removal, whereas the one-stage method is a single surgery). However, in many cases additional operations are required due to the relapse of PJI or other issues (postoperative wound hematomas, spacer dislocations, etc.). The recorded data we worked with contain 15 different types of operations, which were divided into three groups: operations which have no connection with PJI, first case of PJI, or PJI relapse. The full list of operations is presented in Table 3.

### 3.2. Models

#### 3.2.1. Decision Tree

A tree-based imitational model was first used by the authors to study PJI treatment methods in [30]. In that context, a generalized model was introduced, relating the states of the tree to the total number of operations for a given patient, which was considered as a factor for the PJI relapse. It was shown that the functional capacity of a patient is badly affected by repetitive operations, independent of the treatment method. However, the correlation of PJI relapse chance and the number of operations performed were not supported by the data. Following that approach, we developed an algorithm to create and to verify detailed decision trees which distinguish different operation types.

A decision tree describes transitions between the states, which are attributed to different medical interventions. Each state is one of the registered interventions from the patient records database (see Table 3). Each transition signifies the change in patient’s functional capacity and is associated with the treatment costs. The time passed between the transition is not explicitly considered.

An algorithm was developed to build a tree for a given treatment method based on the sample of records for the patients who were treated using this method. The procedure uses a recursive approach and has the following structure:

- For each patient record:
  - Collect the sequence of operations performed;
  - Add the resulting outcome at the end of the sequence as a last patient state, based on death date (if available) and on the PJI status checked during the last observation. Patients who died with confirmed PJI are marked by the state ‘Death’. Those, who had PJI and were alive by the end of the study, are marked by the state ‘Failure’ (of treatment). Finally, those who did not have PJI have the state ‘Success’;
  - Assume that the first state of the decision tree (the root) coincides with the name of the applied treatment strategy, and the second, third, …, n+1-th states are related to the first, second, …, *n*-th registered interventions taken from the patient records (n≤10). The n+2-th state is related to the treatment outcome assigned at the previous step of the algorithm.
- Starting from i=1:
  - Gather the list $L_{i}$ of all recorded intervention types which correspond to the intervention #i in the patient records;
  - Calculate the ratios of occurrence for each operation type $l_{j}^{\left(i\right)}\in L_{i}$;
  - For each $l_{j}^{\left(i\right)}\in L_{i}$:
    * Gather the list $L_{i+1}$ of all recorded intervention types which correspond to the intervention #i+1 in the patient records which had intervention #i equal to $l_{j}^{\left(i\right)}$;
    * Calculate the ratios of occurrence for each operation type $l_{j}^{\left(i+1\right)}\in L_{i+1}$;
    * For each $l_{j}^{\left(i+1\right)}\in L_{i+1}$:
    * …
    * If $L_{k}=\emptyset$, break.

**Figure 1:**
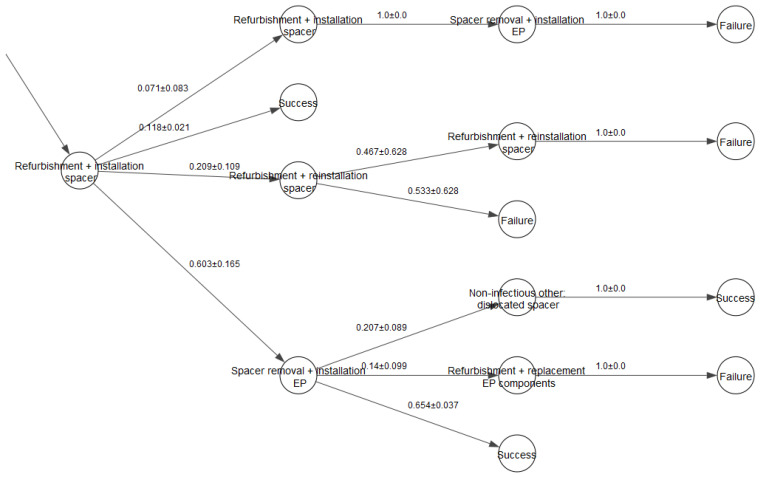
A fragment of the decision tree partial re-THR with confidence intervals for transition probabilities.

A fragment of a decision tree for partial re-THR is shown in Figure 1. The data, which could be derived from the decision tree, include generated individual trajectories and probability distributions for the treatment states calculated via repetitive simulation runs.

#### 3.2.2. Markov Model

A decision tree approach is prone to some issues, particularly:

- It can become intractable if the number of different states and transitions in the patient records is too big;
- It does not consider the time passed between the transitions from state to state.

To address this issues, we developed a Markov model with generalized states as an alternative approach. The detailed description of the model is provided in [31], the major highlights of it are given below.

- To create the model states, the classification described in Table 3, Section 3.1 is used. In addition to the PJI-related interventions (PJI or PJI relapse) and the interventions not related to PJI, a separate intervention type is introduced, which is a second stage intervention for two-stage treatment methods (‘Endoprosthesis installation + spacer removal’);
- The resulting model states are: (a) PJI (waiting for the treatment), (b) second stage (no PJI, waiting for the spacer removal in two-stage treatment methods), (c) additional surgeries (waiting for the treatment of a non-PJI issue), (d) observation (no PJI), and (e) death. The situations of a first PJI case and a recurrent PJI are not distinguished due to the lack of corresponding data in the records;
- The time in the model is discrete, with the time step equal to one month;
- The simulation starts with the state ‘PJI’. The state ‘death’ is an absorbing state.

**Figure 2:**
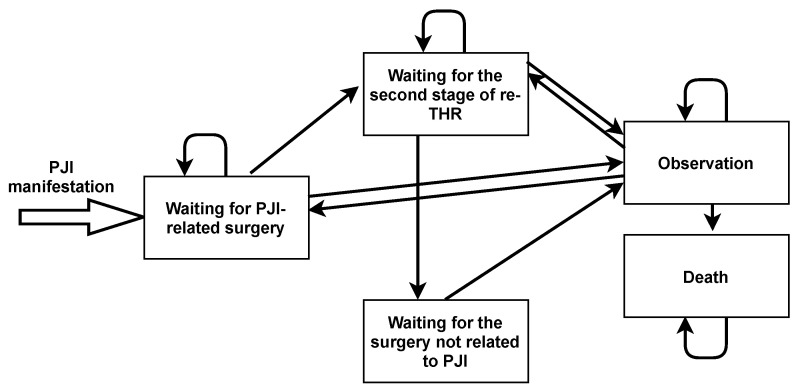
Markov model states and transitions.

The general scheme of state transitions for the model is shown in Figure 2.

The transitional probabilities are calculated based on the available patient records. The calibration procedure consists of the following steps:

- Form a subset of records of patients who were treated using a fixed treatment strategy;
- For each patient, form a list of his subsequent states with the step size of one month, starting from the first manifestation date (i.e., when he was first observed at the hospital with PJI) till the present moment or until he dies. The manifestation dates and intervention dates are used in this process. If the states were changed several times during one month, the last state is taken as the current one at the end of the regarded month;
- Calculate the overall number of transitions between the model states;
- Estimate the transition probabilities via dividing the number of transitions of particular type by the total number of transitions.

The model enables the generation of individual patient trajectories with the consideration of time, which is useful for the calculation of the expected amount of observation time and the hospitalization time.

### 3.3. Model Uncertainty

The accuracy of mathematical modeling depends on how well the mathematical model reflects the properties of the object. The question of special interest is, to what extent a model calibrated on a particular sample is able to predict results for new data. In general, the smaller the training sample is, the bigger is the uncertainty in the model output, and this uncertainty should be quantified to understand the model limitations. In this work, we used a bootstrapping technique to assess the confidence intervals for the transitional probabilities of the models. The procedure of assessing the intervals was the following:

- Draw a random subsample from the patient records database;
- Use the selected subsample to obtain possible model states and calculate the transitional probabilities between them;
- Repeat the procedure *n* times using different subsamples each time;
- Based on obtained samples of size *n*, calculate the confidence interval for each transitional probability using the formula:
  $$\bar{x}-t_{\alpha /2,n-1}\cdot \frac{S}{\sqrt{n}},\bar{x}+t_{\alpha /2,n-1}\cdot \frac{S}{\sqrt{n}},$$
  where $\bar{x}$ is the sample mean, *S* is the sample standard deviation, and $t_{\alpha /2,n-1}$ is a quantile of Student’s *t*-distribution.

### 3.4. Cost-Effectiveness Analysis

To assess and compare the cost-effectiveness of different treatment methods, we implemented algorithms to calculate the statistics of expenses and the overall QALY (quality-adjusted life-years, a generic measure of disease burden), related to different treatment stages. Since the decision trees and the Markov models have different structures, the calculation algorithms, although conceptually similar, differ in some details. The resulting value to measure cost-effectiveness, which is used as an output of the framework, is average cost per QALY for the particular PJI treatment method.

#### 3.4.1. Decision Trees

Since, in a decision tree, each state matches exactly with the particular intervention from the patient record, the intervention costs could be assessed in an easy and straightforward way. At the same time, the time is not tracked in this model type, which makes it complicated to compare time-dependent costs. As it was described in [31], the framework supports the assignments of parameters, related to the impact of the intervention, to each branch of the tree. To assess the cost-effectiveness of the treatment methods, we measured intervention costs in rubles and measured utility of the patient calculated in QALY units. The quantitative outcomes of the treatment in terms of healthcare costs and QALY units gained by the patient might be derived from the decision tree using the following formula:

(1) $$C=\sum_{i}p_{i}\cdot c_{i},\;$$

where $p_{i}$ is the probability of selecting the branch, obtained by cross-validation, $c_{i}$ is the impact measured in either of the two units. The interval assessment of *C* can be calculated using the same formula with left and right boundaries for $p_{i}$ used instead of their mean assessments.

The resulting values of $C_{rubl}$ and $C_{u}$ are used to calculate the costs of one QALY unit and analyze them for different treatment strategies. To calculate QALY units and costs for particular tree branches, we relied on the data provided by Russian Scientific Research Institute of Traumatology and Orthopedics named after R.R. Vreden. The operation costs were taken from the disease histories, and the QALY units were assessed based on the EQ-5D indices for each particular patient measured between the subsequent operations according to the methodology described in [32].

#### 3.4.2. Markov Models

The treatment impact for a fixed individual patient trajectory is calculated according to the formula

(2) $$C\left(t\right)=\sum_{i}t_{i}\cdot \bar{c_{i}},\;$$

where $\bar{c_{i}}$ are the monthly costs or QALY units associated with the patient state *i* in the model, and $t_{i}$ is the expected average patient’s time of staying in a state *i* (the number of months). Due to the fact that in the Markov model we use generalized patient conditions which are not tied to particular intervention types, the accurate values for $\bar{c_{i}}$ (both in rubles and QALY units) cannot be found in records. The expenses for every model state were assessed by averaging the costs of all possible interventions associated with that particular state, and the QALY units gained were found based on the experts’ opinion. We assumed that the patient gains maximum QALY units when he is in the ‘Observation’ status. The lowest QALY values correspond to ‘PJI’. The quality of life of a patient waiting for the second stage or additional surgeries is higher than in case of PJI, but lower than in the ‘Observation’ state due to corresponding health issues (particularly, the patients waiting for the second stage of the treatment have limited mobility due to spacer installation which badly affects their QALY count). Under the expert assumption, we assumed the QALY for the ‘PJI’ state equal to 0.35, for ‘Second stage’ equal to 0.7, for ‘Non-PJI operation’ equal to 0.5, for ‘Observation’ equal to 0.85, and for ‘Death’ equal to 0.

As an example, we consider a patient who undergoes a two-stage therapy with a three-month interval between stages. The model presents the chain of states ‘Waiting for surgery related to PJI (month 1)’, ‘Waiting for the second stage of therapy’ (month 2), ‘Waiting for the second stage of therapy’ (month 3), ‘Waiting for the second stage of therapy’ (month 4), and ‘Observation’ (month 5). When searching for the average QALY values obtained with various methods of therapy, based on the Markov model, the length of stay in each state is multiplied by the QALY units characteristic of it. In our example, we have to sum 1 × 0.35 (QALY for PJI condition, 1 month duration), 3 × 0.7 (QALY for the period of waiting for the second stage, 3 months duration) and 1 × 0.85 (observation, 1 month duration).

For calculating costs, along with the cost of staying in a model state during a certain amount of time (as in QALY calculation), the cost of operations should also be considered. In the setting of generalized Markov model operations are attributed to transitions between states. For instance, a transition from ‘Waiting for the second stage of therapy’ to ‘Observation’ implies a performed operation with spacer removal and endoprosthesis installation. Consequentially, in the above example, the total cost will consist of the following terms:

- The cost of a month of inpatient stay awaiting surgery related to PJI (state of the model);
- Cost of the PJI operation (the transition of the model from “Waiting for surgery with PJI” to “Waiting for the second stage of therapy with PJI”);
- Cost of three months of waiting for the second stage of therapy (state of the model);
- Cost of the operation of the second stage of therapy with PJI (transition of the model from “Waiting for the second stage of therapy with PJI” to “Observation”);
- The cost of a month in the “Observation” state.

The results of assessing cost-effectiveness of PJI treatment using two described modeling approaches are presented in the following section.

## 4. Results

An algorithm for model calibration and cost-effectiveness assessment was implemented using Python programming language, with *numpy*, *pandas*, and *scipy* libraries employed for data management and *python-igraph* library employed to draw decision trees. For each considered treatment method, a separate decision tree and a Markov model were built. In both cases all possible disease states related to a particular treatment were established and transition probabilities between the model states were assessed in a form of confidence intervals for mean values. Confidence intervals were calculated through bootstrapping based on 10 subsamples from the patient record database, with a size of every subsample equalled 80% of the whole database. For the sake of saving space, we omitted resulting decision trees. Resulting Markov models for all the treatment methods with calculated transitional probabilities are presented in Appendix A.

The values of QALY and costs were calculated as sums during a fixed period of time after the first case of PJI (24 months for decision trees and 30 months for Markov models). The results along with the costs per QALY are shown in Table 4 and Table 5 (for retrospective methods) and Table 6 and Table 7 (for prospective methods). The optimal values (higher QALY, lower costs) are marked in bold.

It is important to mention that due to differences in assessing both QALY and costs, the corresponding values of these characteristics cannot be compared between the two employed models (decision trees and Markov models). The comparison that could however be made is related to the ranks of treatment types related to their cost-effectiveness (smallest cost per QALY, second smallest, etc.). From this perspective, different modeling methods agree on the fact that RA (resection arthroplasty) is the optimal treatment method among the retrospective methods. In case of comparing treatment methods used for the prospective cohort of patients, the results are not so consistent, as the tables clearly demonstrate.

## 5. Discussion

In this paper, a modeling framework is presented which aims at facilitating the decision-making for the healthcare professionals in the area of periprosthetic joint infection treatment. By using two different approaches within one framework, one can obtain a detailed static analysis of the prospected patient treatment trajectories, depending on the selected strategy (decision tree approach), or, alternatively, perform a dynamic simulation of a patient trajectory of transitions between the generalized states in an imitational model (Markov modeling approach). The former helps to calculate detailed total operational costs and quality of life obtained, e.g., their average values and their distributions, whereas the latter offers an opportunity to dynamically monitor and forecast the dynamics of costs and QALY units. In the current study, we demonstrated how the framework could be used to compare different treatment methods based on the following indicators:

- Total/average treatment impact related to the increase in quality of life for the patient (in QALY units) and the operational costs for the healthcare unit (in rubles);
- Proportions between the costs in rubles and the utility gained for one average patient or a group of patients (costs of one QALY unit, or costs per QALY).

Both model types within the framework demonstrate a compromise between the explanatory and predictive power of the model. Particularly:

- The approach connected with the decision trees makes it possible to trace the sequence of operations in high detail, thus making it easier to accurately calculate average QALY and costs per model state, since one state is easily interpreted as an actual medical procedure. At the same time, detailed states make it harder to use the model for prediction purposes. The limited sample sizes for particular treatment methods dramatically increase the uncertainty in transition probabilities assessment and the nomenclature of possible states themselves. Lastly, since the model is event-based and does not include time, it is not suitable for dynamic time-explicit prediction of the health outcomes. Only the ultimate result for the patient might be established (PJI-related death or death from other causes).
- In comparison with decision trees, the Markov model is better suitable for handling treatment processes based on small patient samples due to its generalized states. Additionally, it is more suitable for prediction of individual patient trajectories. Since the Markov model includes time, it allows to monitor time-related costs and expenses. The drawbacks of the model include complications in calculating QALY and costs per state. The generalized states have somewhat abstract interpretation and, therefore, some form of averaging is inevitable in calculating $\bar{c_{i}}$ (Formula (2)), which increases the calculation uncertainty.

We assume that for thorough cost-effectiveness analysis, both modeling methods could be applied in parallel to mutually compensate their weak spots.

It is also important to mention that the cost-effectiveness analysis results in an outcome demonstrated in Table 4, Table 5, Table 6 and Table 7, which cannot serve as a direct guide to action related to the selection of the treatment method for the particular patients, because the regarded treatment methods cannot be considered totally interchangeable. In addition to the cost-effectiveness, there are other factors that influence method selection, among which are individual conditions of the regarded PJI case and personal characteristics of patients which might favor one or another treatment method. To compensate this drawback, we suggest using more patient parameters from the database and developing the treatment models based on patients’ individual characteristics (age, gender, body mass index) as parameters affecting the transition probabilities. In this case, a calculation of individual treatment trajectories becomes possible, which converts the described framework into a more powerful software tool within the personalized medicine approach.

In addition to the mentioned framework modification, we consider another way of framework development which includes the changing of the modeling scale. We propose a framework evolution towards a geographically explicit prediction of dynamics of PJI cases in time at a city level, using synthesized populations as a model input [33,34]. The modified modeling framework for the PJI treatment can be fed by synthetic data coming from a demographic model for the urban population and a statistical model of PJI occurrence in that population. As a result, it will become possible to assess the hospital occupancy, the long-term consequences of PJI treatment, and the prospected potential years of life lost in the urban population depending on the prevalent treatment methods.

## Figures and Tables

**Table 1:** Demographic characteristics of study participants.

Treatment	Age/Sex	<20	21–30	31–40	41–50	51–60	61–70	71–80	81–90	>90	Total
2-stage > 2 mth	M		3	20	33	59	50	26	5		196
	F	1	2	17	17	46	46	36	6		171
1-stage retro	M								1		1
	F							1	1		2
2-stage 2–3 wk	M			2	4	1	3	1			11
	F					1	1	1			3
2-stage 6–8 wk	M			8	8	9	6		1		32
	F				2	5	10	2			19
1-stage	M			1	4	4	11	9	3	1	33
	F				1	6	14	13	11		45
RA	M				1	2	4		1		8
	F				1		2	2			5
re-THR-PE	M		1	4	2	5	3	3	2		20
	F			1	3	10	9	8			31
Partial-I	M										
	F			1	1	3	2	1	3		11
Partial-II	M			2	3			2			7
	F			1	2		2				5
**Total**		1	6	57	82	151	163	105	34	1	600

**Table 2:** Description of treatment methods.

Abbreviation	Full Name	Description
re-THR-PE	Revision operation with the preservation of endoprosthesis	The joint is opened and washed. The parts of the artificial joint, which can be easily removed, are replaced with the new ones, and the wound is closed.
2-stage re-THR	Two-stage total hip replacement with > 2 months (2–3 weeks, 6–8 weeks) between the stages	The joint is opened and cleaned up, an antibacterial spacer is placed, and the wound is closed. After a certain time period, the joint is opened again, the spacer is removed, the prosthesis is installed and the joint is closed.
RA	Resection arthroplasty	The joint is opened, everything is removed, the hole in the tissues is filled with a muscle cut from the thigh, and the wound is closed.
1-stage	One-stage total hip replacement	The joint is opened, everything is removed, a new endoprosthesis is installed and the wound is closed.
Partial-I	Partial one-stage total hip replacement	Equal to one-stage re-THR, but with partial preservation of the endoprosthesis.
Partial-II	Partial two-stage total hip replacement	Equal to two-stage re-THR, but with partial preservation of the endoprosthesis.

**Table 3:** Operations performed and their relation to PJI.

No PJI	First Case of PJI or PJI Relapse	PJI Relapse
Endoprosthesis (EP) installation + spacer removal;	Debridement + spacer installation;	Debridement + spacer reinstallation;
EP installation (no spacer);	Debridement;	Disarticulation;
Non-infectious: spacer dislocation;	EP components replacement + debridement;	Spacer removal + support osteotomy;
Other: (suturing, etc.);	Debridement + full EP replacement;	Debridement + support osteotomy + muscle plastic;
Non-infectious: periprosthetic fracture case;	Joint drainage + long-term suppressive antibiotic therapy (ABT);	Joint drainage

**Table 4:** Average costs for treatment methods in the retrospective group according to the decision trees.

	re-THR-PE	2-Stage > 2 Months	RA
QALY	2.08	4.19	**6.30**
Cost, rubles	142,367	239,770	**90,220**
Costs per QALY, rubles	68,411.22	57,200.29	**14,323.45**
Rank of effectiveness	3	2	**1**

**Table 5:** Average costs for treatment methods in the retrospective group according to the Markov model simulations.

	re-THR-PE	2-Stage > 2 Months	RA
QALY	1.88	**1.92**	1.79
Cost, rubles	117,634	243,670	**105,920**
Costs per QALY, rubles	62,571.27	126,911.46	**59,173.18**
Rank of effectiveness	2	3	**1**

**Table 6:** Average costs for treatment methods in the prospective group according to the decision trees.

	2-Stage 2–3 wk	2-Stage 6–8 wk	1-Stage	Partial-I	Partial-II
QALY	8.16	**8.89**	3.21	4.59	8.6
Cost, rubles	314,771	289,315	**144,815**	158,484	264,606
Costs per QALY, rubles	38,596.55	32,562.1	45,095.1	34,546.21	**30,766.29**
Rank of effectiveness	4	2	5	3	**1**

**Table 7:** Average costs for treatment methods in the prospective group according to the Markov model simulations.

Treatment Method	2-Stage 2–3 wk	2-Stage 6–8 wk	1-Stage	Partial-I	Partial-II
QALY	1.925	**2.055**	1.99	1.83	2.0
Cost, rubles	288,507	300,471	147,687	**135,370**	267,436
Costs per QALY, rubles	149,874.77	146,214.6	74,214.57	**73,972.68**	133,718
Rank of effectiveness	5	4	2	**1**	3

**Figure A1:**
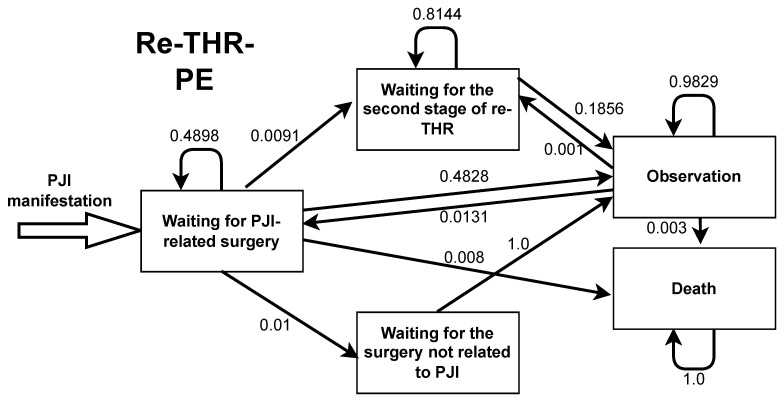
Markov model transition probabilities for revision operation with the preservation of endoprosthesis.

**Figure A2:**
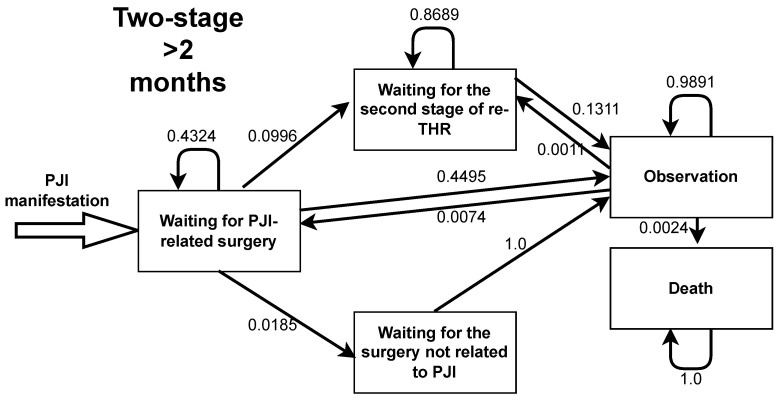
Markov model transition probabilities for two-stage total hip replacement with > 2 months between the stages.

**Figure A3:**
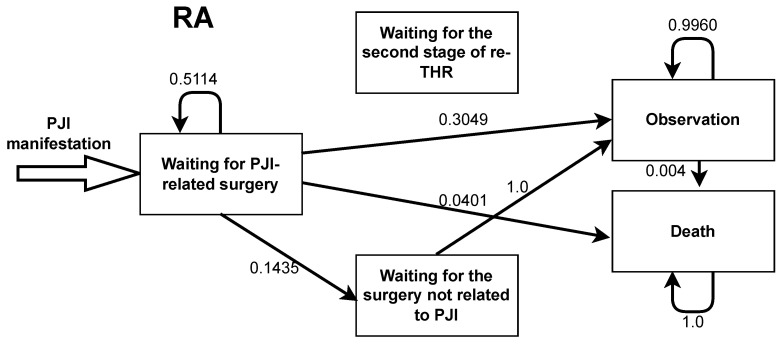
Markov model transition probabilities for resection arthroplasty.

**Figure A4:**
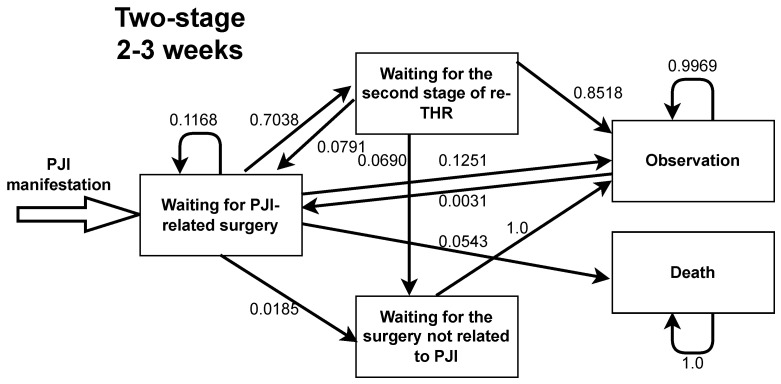
Markov model transition probabilities for two-stage total hip replacement with 2–3 weeks between the stages.

**Figure A5:**
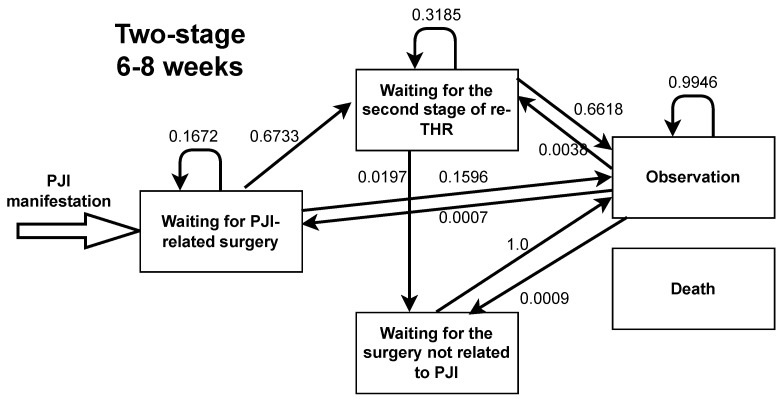
Markov model transition probabilities for two-stage total hip replacement with 6–8 weeks between the stages.

**Figure A6:**
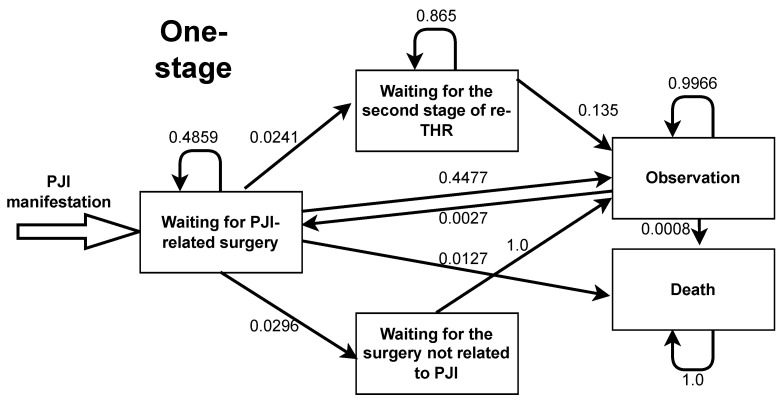
Markov model transition probabilities for one-stage total hip replacement.

**Figure A7:**
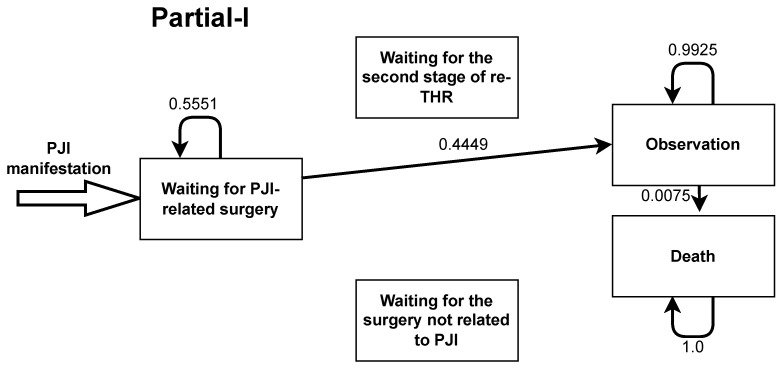
Markov model transition probabilities for partial one-stage total hip replacement.

**Figure A8:**
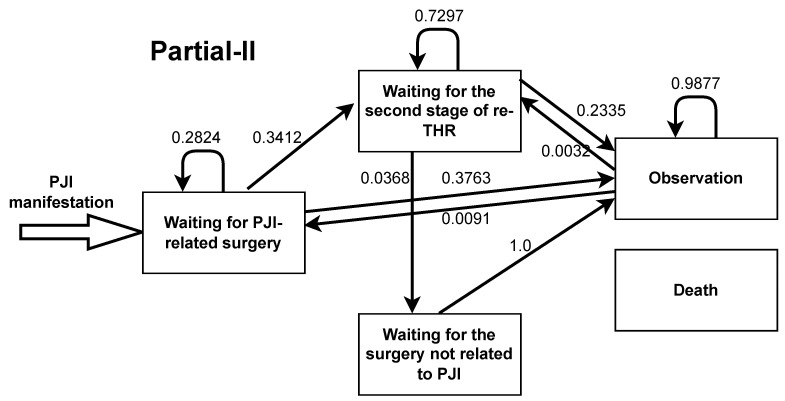
Markov model transition probabilities for partial two-stage total hip replacement.

## Data Availability

Data available upon request.
